# modelBuildR: an R package for model building and feature selection with erroneous classifications

**DOI:** 10.7717/peerj.10849

**Published:** 2021-02-09

**Authors:** Maximilian Knoll, Jennifer Furkel, Juergen Debus, Amir Abdollahi

**Affiliations:** 1 Department of Radiation Oncology, Heidelberg University Hospital, Heidelberg, Deutschland; 2 National Center for Tumor Disease (NCT), UKHD and German Cancer Research Center (DKFZ), Heidelberg, Germany; 3 German Cancer Consortium (DKTK), Core Center Heidelberg, DKFZ, Heidelberg, Germany

**Keywords:** Feature selection, Misclassification, Model building, Ground truth, High dimensional data, Glioblastoma multiforme, Prognosis, Long term/short term survivor, Illumina humanmethylation array data, G-CIMP negative GBM

## Abstract

**Background:**

Model building is a crucial part of omics based biomedical research to transfer classifications and obtain insights into underlying mechanisms. Feature selection is often based on minimizing error between model predictions and given classification (maximizing accuracy). Human ratings/classifications, however, might be error prone, with discordance rates between experts of 5–15%. We therefore evaluate if a feature pre-filtering step might improve identification of features associated with true underlying groups.

**Methods:**

Data was simulated for up to 100 samples and up to 10,000 features, 10% of which were associated with the ground truth comprising 2–10 normally distributed populations. Binary and semi-quantitative ratings with varying error probabilities were used as classification. For feature preselection standard cross-validation (V2) was compared to a novel heuristic (V1) applying univariate testing, multiplicity adjustment and cross-validation on switched dependent (classification) and independent (features) variables. Preselected features were used to train logistic regression/linear models (backward selection, AIC). Predictions were compared against the ground truth (ROC, multiclass-ROC). As use case, multiple feature selection/classification methods were benchmarked against the novel heuristic to identify prognostically different G-CIMP negative glioblastoma tumors from the TCGA-GBM 450 k methylation array data cohort, starting from a fuzzy umap based rough and erroneous separation.

**Results:**

V1 yielded higher median AUC ranks for two true groups (ground truth), with smaller differences for true graduated differences (3–10 groups). Lower fractions of models were successfully fit with V1. Median AUCs for binary classification and two true groups were 0.91 (range: 0.54–1.00) for V1 (Benjamini-Hochberg) and 0.70 (0.28–1.00) for V2, 13% (*n* = 616) of V2 models showed AUCs < = 50% for 25 samples and 100 features. For larger numbers of features and samples, median AUCs were 0.75 (range 0.59–1.00) for V1 and 0.54 (range 0.32–0.75) for V2. In the TCGA-GBM data, modelBuildR allowed best prognostic separation of patients with highest median overall survival difference (7.51 months) followed a difference of 6.04 months for a random forest based method.

**Conclusions:**

The proposed heuristic is beneficial for the retrieval of features associated with two true groups classified with errors. We provide the R package modelBuildR to simplify (comparative) evaluation/application of the proposed heuristic (http://github.com/mknoll/modelBuildR).

## Introduction

Model training is an important task in biomedical research for the evaluation of omics data, e.g., for classification tasks. The features included in the model and used for classification might hint towards underlying (biological) processes or mechanisms.

Such classifications in biomedical research are often encoded by a human rater as binary, e.g., a given immune-histochemistry staining can be classified as positive/negative (1/0), or as semi-quantitative score (e.g., 0–5) for graduated evaluation (Balermpas et al., 2017; Knoll et al., 2016). Often, associated changes on molecular level are of interest, measured e.g., by analysis of expression or methylation data with arrays/sequencing yielding a high number of features.

Binary outcome data can be modeled using a logistic regression, a generalized linear model (GLM) with logit link function (Hastie & Tibshirani, 1986; McCullagh & Nelder, 1989). Semi-quantitative data might be evaluated using linear models.

For model training, a full evaluation of all feature combinations is usually not feasible (high number of features), and standard GLMs cannot be trained for numbers of features >numbers of observations, requiring the usage of heuristics for pre-filtering of features. A set of remaining features can then be used to train a model, e.g., using backward selection in combination with an information criterion (Akaike, 1973).

Model fits are usually evaluated for their ability to predict the observed data (“goodness-of-fit”). The latter might, however, contain erroneous assignments, arising e.g., from multiple sources (technical difficulties, sampling or human error). Thus, forcing the model to fit the observed rather than the true underlying groups might lead to the selection of inappropriate features.

We therefore propose to use a heuristic for feature pre-filtering prior to model building which reverses the role of dependent (classification)/independent (features) variables, perform tests for difference and cross validate data with reverted roles of dependent/independent variables, and use only retained features for subsequent model building.

Its performance is compared to a standard cross validation approach (non-reverted roles of variables) in simulated data. Binary and semi-quantitative encodings (with added errors) in features sampled from two or more populations are evaluated, and the ability of both approaches to select meaningful features (high overlap with known ground truth).

We provide an R package to simplify (comparative) analyses with the proposed heuristic, available on github (http://github.com/mknoll/modelBuildR).

## Methods

### Feature selection methods

**Figure 1:**
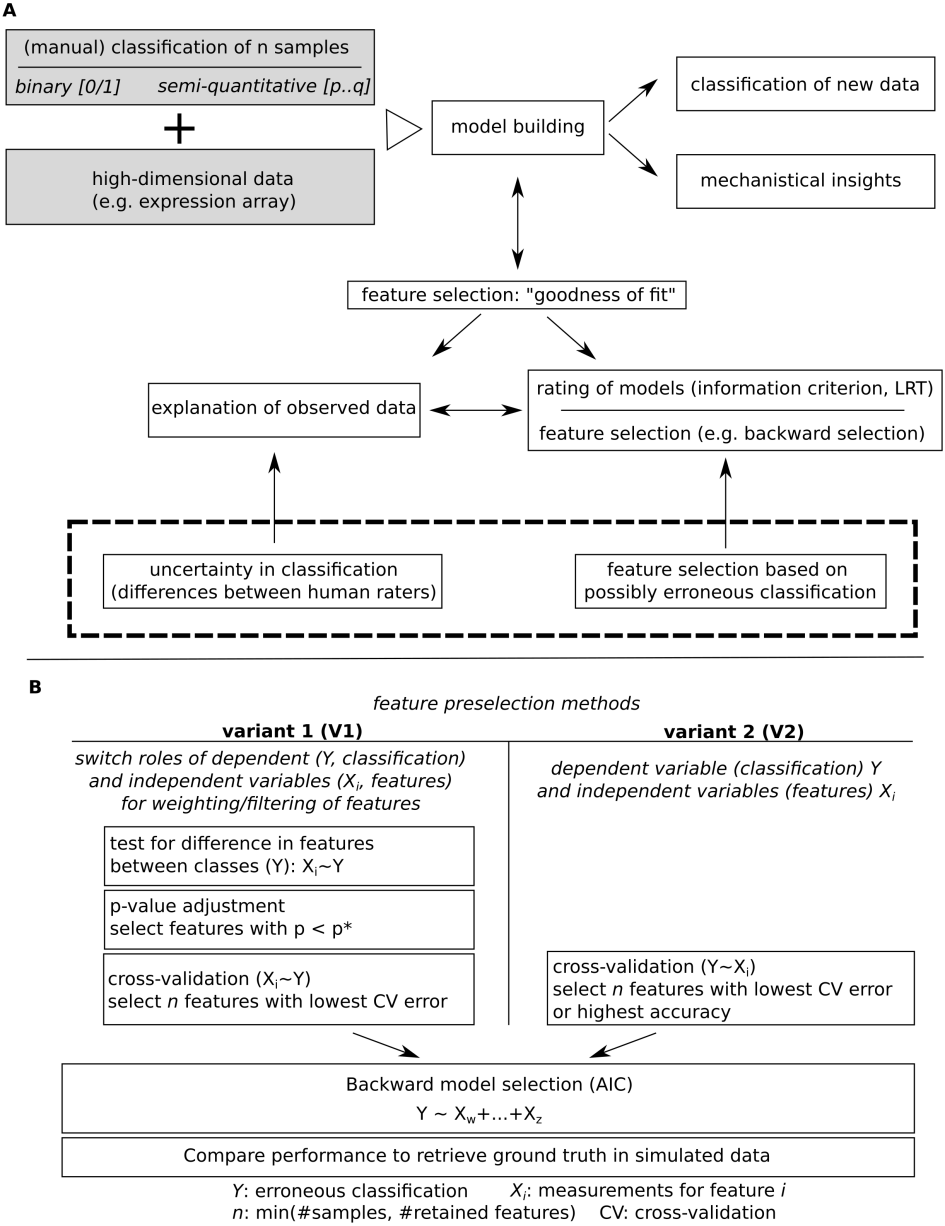
Model building for a given classification using high dimensional data. (A) Major steps in model building and outline of the subsequently addressed issue of potential erroneous classification. (B) Comparatively evaluated strategies for feature pre-filtering/ordering of features prior to model building and outline of how model performance is evaluated.

**Figure 2:**
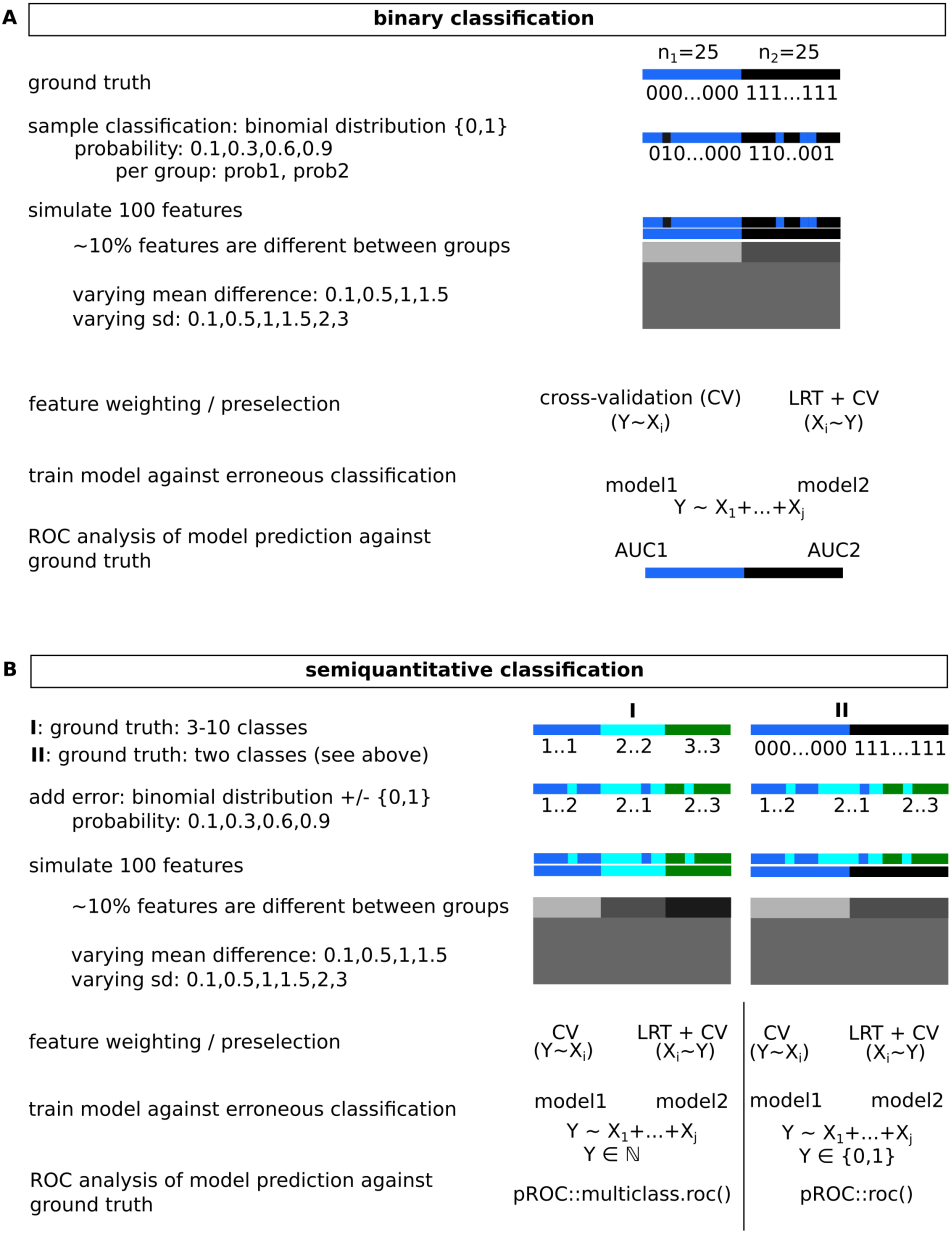
Simulated data for binary (A) and semi-quantitative classification (B) and the introduction of errors used for evaluation of feature selection/model building approaches. Observed classification was sampled from a binomial distribution for varying probabilities per group, for semi-quantitative data, equidistance between classes was assumed, and errors were added based on data sampled from binomial distributions.

**Figure 3:**
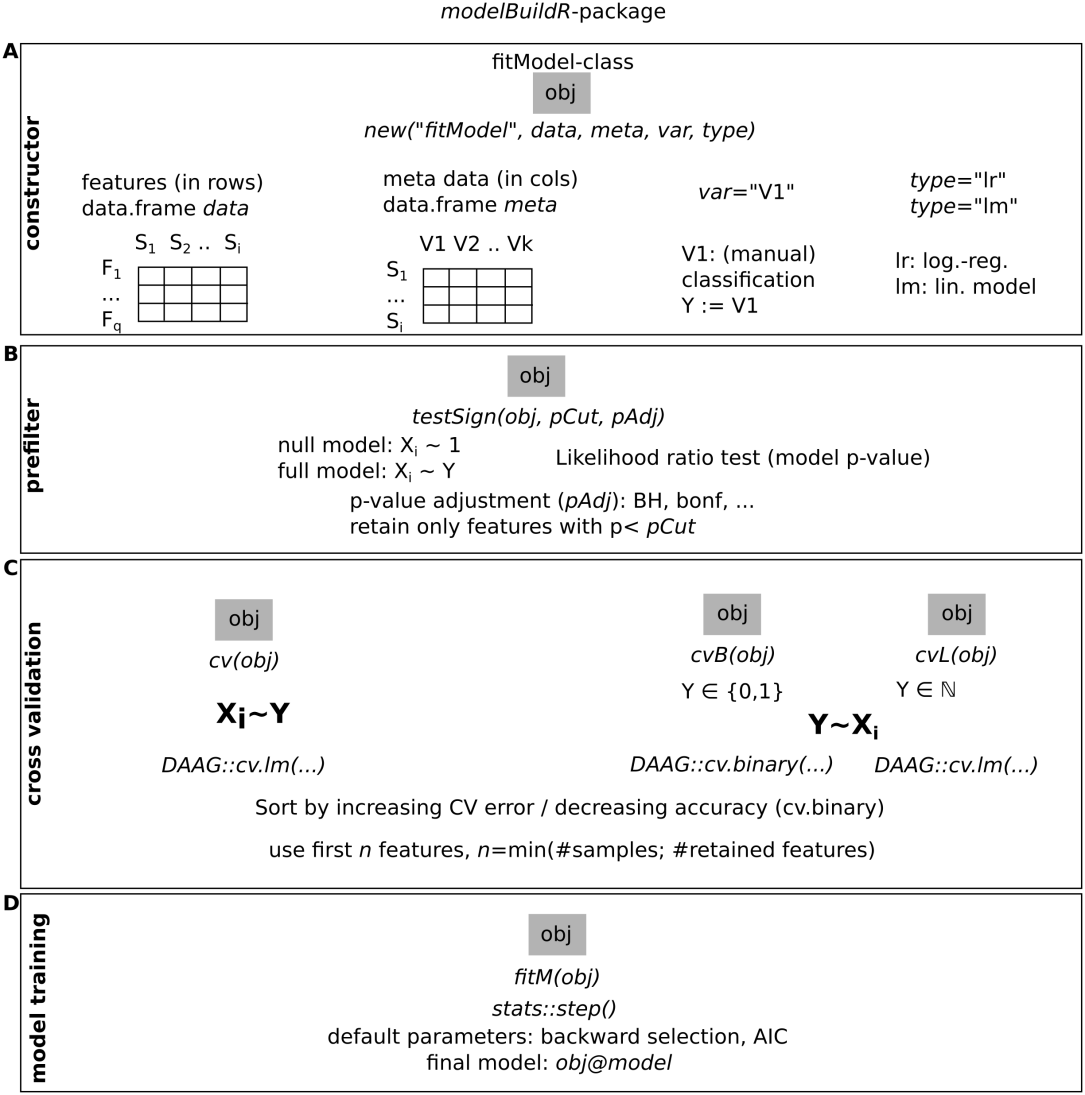
Overview of the functions implemented in the modelBuildR package and required parameters. (A) Instantiation of a fitModel object for analysis. (B) Prefiltering of features. (C) Cross-validation approaches. (D) Final model fitting.

The two evaluated feature selection methods are outlined in Figs. 1B and 2. Variant 2 (V2) uses cross validation to obtain an order on single features (univariate test), using the (erroneous) classification as dependent variable. For binary outcomes, cv.binary() from the DAAG package (Maindonald & Braun, 2020) and for semi-quantitative outcomes, cv.lm() was used (default parameters, Fig. 3). The first n features with lowest cross-validation errors or highest accuracy were selected for model selection, with *n* being the number of evaluated samples (here: 50). Further processing was similar between both evaluated methods. Variant 1 inverts the role of dependent (classification)/independent (features) variables for the initial feature filtering step. First, a significant influence of the observed classification on each measured feature is tested using a linear model and calculating model *p*-values (null- vs full models, likelihood ratio test, LRT). *P*-values are then adjusted for multiplicity, Benjamini–Hochberg and Bonferroni adjustment was evaluated, all features with adjusted *p*-values below 0.05 (*p*∗ = 0.05, Fig. 1B) were retained. Next, a cross validation step was performed, keeping the inverted roles of independent/dependent variables. Finally, the first n features with lowest cross-validation errors were used for further analysis, with n being the minimum of the number of remaining features and numbers of samples. For the next step, which is similar to variant 2, the original roles of the dependent and independent variables were assumed (classification: dependent variable). Model building was performed by backward model selection using AIC, with a logistic regression for binary outcomes and a linear model for semi-quantitative classification. Predictions were then compared to known underlying group truth by calculation AUCs with pROC::roc() (Robin et al., 2011) for binary and pROC::multiclass.roc() for semi-quantitative classifications (Fig. 2). AUCs and AUC ranks (tie methods: average, random) were evaluated.

### Evaluated data

An overview of simulated data gives Fig. 2. Two common cases were tested, a binary classification and a semi-quantitative graduated classification together with high-dimensional data. To keep calculation time reasonable, a total number of 100 features was evaluated in 50 samples. Only a fraction of features (∼10%, sampled with runif()) were assumed to show differences between groups. For two group analyses, they were sampled from two normal distributions with varying differences in means and standard deviations. For more than two classes, differences between means of subsequent classes/distributions were constant, as were their standard deviations. Group sizes were balanced, if this was not possible, the sample number of the highest ranked group was expanded. To assure reproducibility, a fixed seed was used. Errors on the classifications were introduced as follows: for a binary classification, the respective group assignment was retrieved from a binomial distribution yielding 0,1 with probabilities prob1 and prob2. For larger numbers of classes, a vector of 0,1 values was obtained similarly for a probability prob1, and was subtracted from the true classification. Absolute values were used as erroneous classification. Reproducible code and analyses are available as CodeOcean capsule: https://codeocean.com/capsule/3333162/tree/v1.

### modelBuildR package

The presented analyses were performed with the modelBuildR package. An overview of its functionality is shown in Fig. 3, additional functionality is outlined in the package vignette.

The constructor for a new fitModel instance requires a feature data.frame data with features in rows and samples in colums, a metadata data.frame meta with samples in rows and covariates in columns, specification of the classification (dependent) variable var and the type of model to train (type, lr for logistic regression and lm for linear model). The evaluation of an association of the classification variable on feature measurements is performed with testSign(), expecting a multiplicity adjustment parameter (pAdj, all allowed methods from stats::p.adjust()) and a *p*-value cutoff (pCut). Different cross-validation methods are implemented, cv() performs the cross validation on inverted roles of dependent/independent variables as described above (Figs. 1B and 2). cvB() and cvL() perform cross-validation on non-inverted roles of variables. fitM() finally performs the model training using R’s stats::step() function with default parameters (using AIC or BIC) or using a cross-validation approach (see Suppl. Methods for details).

In addition to previously outlined analyses, the feature preselection step can also be performed while including additional covariates (both for the model evaluation and cross validation step, refer to the package vignette for details: vignette(“modelBuildR”)).

### Omics data and alternative feature selection methods

450k Illumina human methylation array data and clinical information of the TCGA-GBM cohort was retrieved through the GDC data portal on 2019-11-07. Logit transformed methylation data was used for analysis if not stated otherwise (*M* values). G-CIMP classification was performed as follows: *L* = 282.7+114.2*cg06903384, *p* = exp (*L*)/(1 + exp (*L*)). Samples with *p* < 0.5 were classified as CIMP- and CIMP+ otherwise. The glmnet package (Friedman, Hastie & Tibshirani, 2010) was used for lasso regression, utilizing cross validation to select an appropriate lambda value, and randomForest (Liaw & Wiener, 2002) for random forest analysis. Student’s *t*-tests were used. Optimal cutoffs of prognostic separation (minimal *p*-value) were calculated with dataAnalysisMisc::findOptCutoff() (dataAnalysisMisc, 2020). The pvclust package (pvclust, 2019) was used for consensus clustering, the umap R package in combination with umap-learn for dimensionality reduction (Konopka, 2020; McInnes & Healy, 2018). Significance level alpha was fixed at 0.05 (two-sided).

## Results

### Semi-quantitative classification and true graduated differences in underlying data

**Figure 4:**
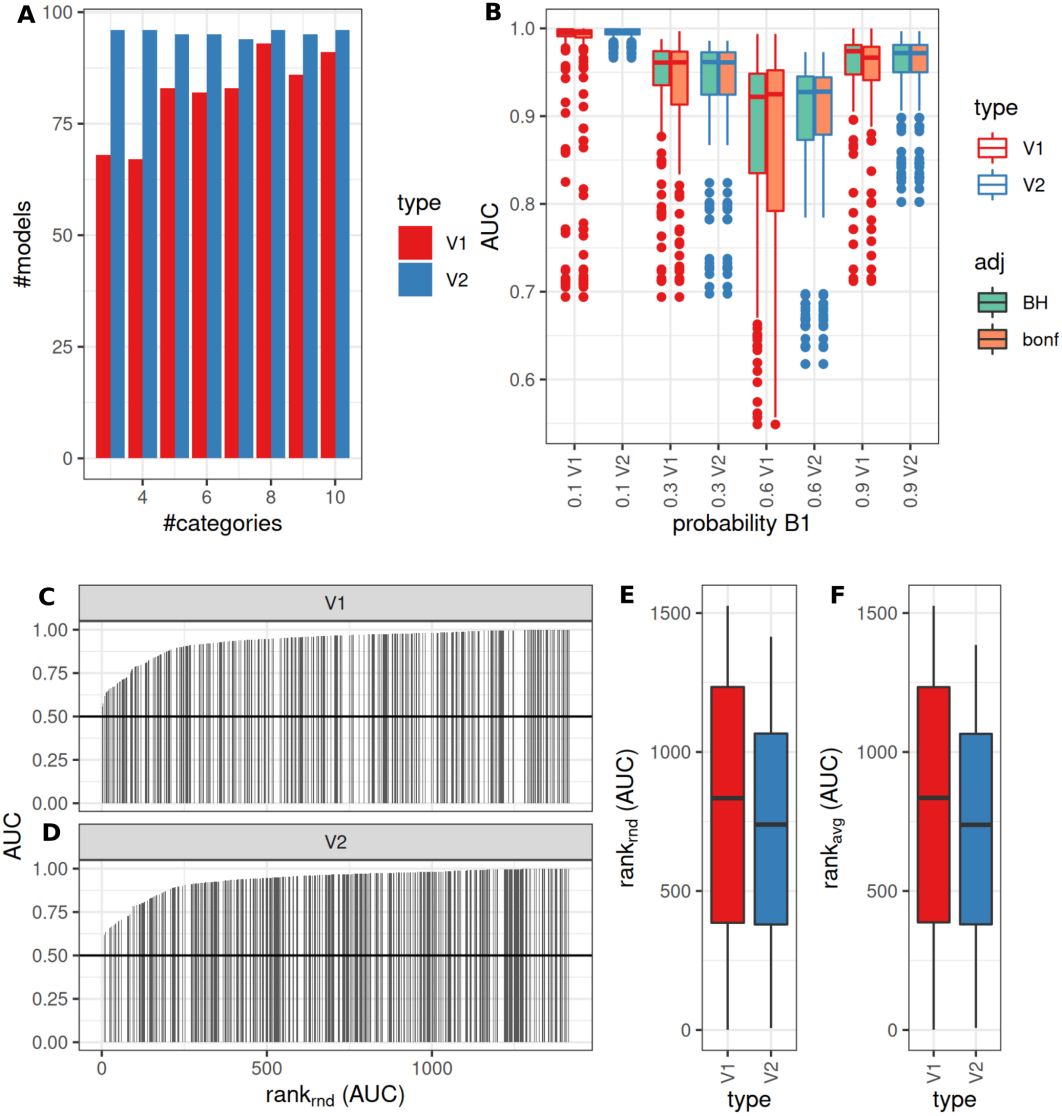
Comparison of feature preselection methods for semi-quantitative classification with true underlying equidistant differences between groups. (A) Number of successfully trained models (V1, Benjamini-Hochberg multiplicity adjustment). (B) AUCs of model predictions tested against ground truths for varying classification errors and different multiplicity adjustment methods. AUCs (C–D) and AUC ranks (E–F) of V1 and V2, rank-ties methods: avg, average, rnd, random.

Model fitting (>0 as significantly different identified features, *pAdj* < 0.05, Benjamini–Hochberg adjustment) was successful more frequently when using V2. V1 showed lower fractions for fewer categories (successful model fits, reference V2: median: 88%, range: 70–97%, Fig. 4A). Observed median AUCs were similar between V1 and V2 (Fig. 4B) and did not differ between *p*-value adjustment methods (V1, Fig. 4B). AUCs stratified by numbers of true underlying groups and mean difference for BH and Bonferroni adjustments are shown in Figs. S2 + S3. Differences between V1/V2 of observed AUCs decreased for increasing numbers of categories, single outliers were observed mostly for V1. Higher uncertainty in classification (prob1 0.3/0.6) showed lower AUCs especially for fewer groups (Figs. S2 + S3). Larger standard deviations with smaller mean differences decreased AUCs for V1, more prominent for Bonferroni than for Benjamini–Hochberg adjustment (Figs. S2 + S3). Median model fitting time requirements were lower for V1 (Fig. S1).

### Binary classification and two true underlying groups

**Figure 5:**
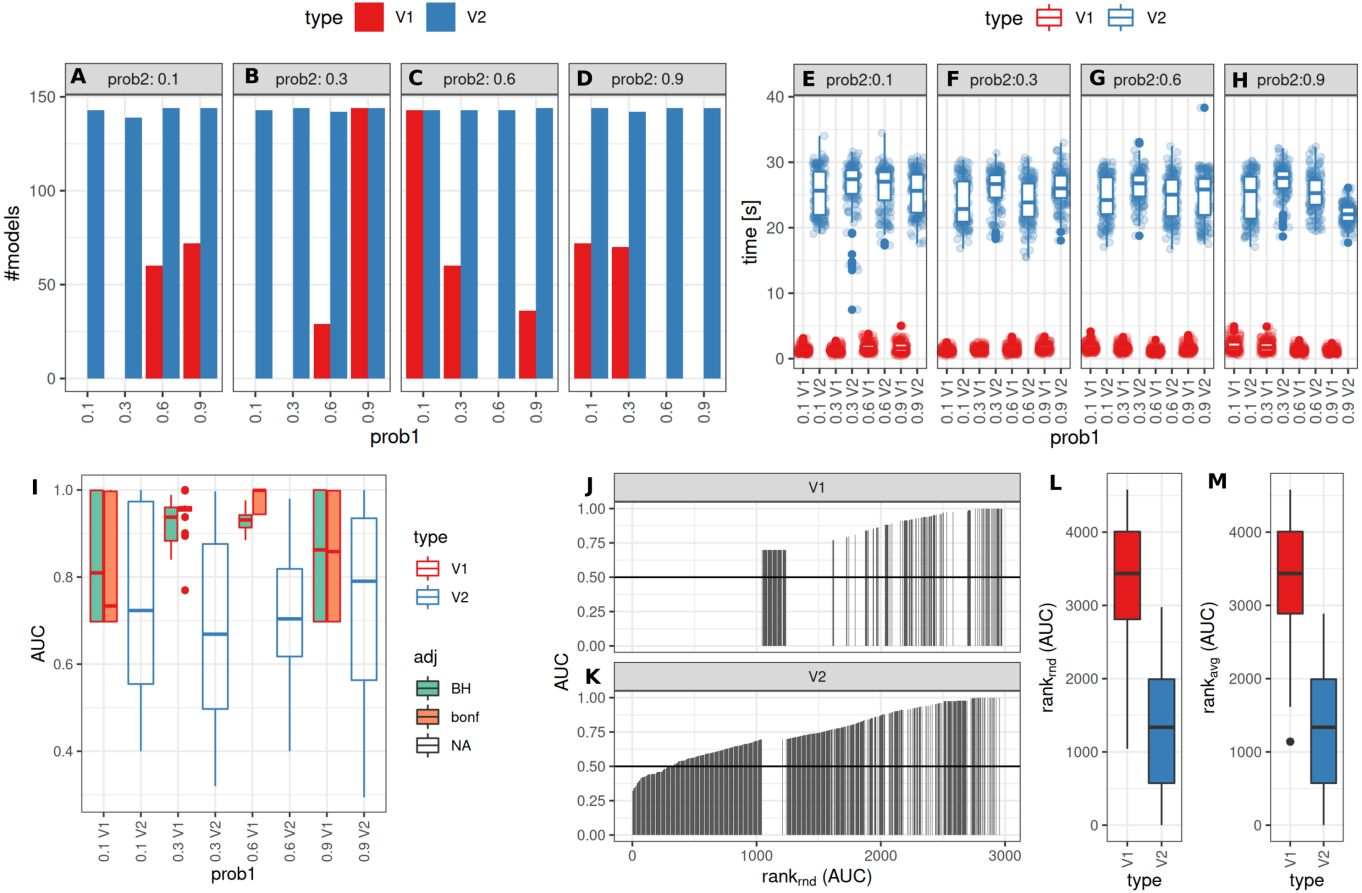
Comparison of feature preselection methods for binary outcomes with underlying true dichotomous groups. (A–D) Number of successfully trained models (Benjamini-Hochberg multiplicity adjustment). (E–H) Time requirements for model fitting. (I, J, K) AUCs of model predictions tested against ground truths for varying classification probabilities. (L–M) AUC ranks for V1 and V2, rank-ties methods: avg, average, rnd, random.

The number of successful model fits ranged between 139 and 144 for V2 and 0 and 144 for V1 (Benjamini–Hochberg adjustment, Fig. 5A). Processing times were lower for V1 (<5 vs >20 s, Fig. 5B, Fig. S1). Minimum observed AUCs were >0.5 for all combinations evaluated with V1, 13% of models lead to an AUC < = 0.5 for V2 (Fig. S5). Separate analysis for combinations of prob1/prob2 showed that 0.1/0.1; 0.3/0.1; 0.1/0.3; 0.3/0.3; 0.6/06; 0.6/0.9; 0.9/0.9 did not yield any models for V1 (Fig. 5A), V2 identified models with low AUCs in these cases (Figs. S4 + S5). No general difference in AUCs between Bonferroni and Benjamini–Hochberg adjustment could be detected when separating results by probability (Figs. S4 + S5). Increasing mean differences allowed model fitting for larger standard deviations with V1, with higher median AUCs for Bonferroni adjustment for most evaluated combinations (Figs. S4 + S5, Fig. 5C). Higher median AUC ranks were observed for V1 (Fig. 5D), as well as higher median AUCs (Fig. 5C).

Performance of both approaches were additionally tested for larger numbers of features (up to 10,000) and higher number of samples per group (same size, up to 100) and with probabilities 0.1 and 0.3 with Bonferroni *p*-value adjustment. Results are shown in Fig. S7. For *n* = 25 samples per group, no models could be fitted with V1. Both AUCs and AUC ranks were higher for V1, up to AUCs of 1 where the corresponding models from V2 reached AUCs not above 0.8. Minimum observed AUCs for V1 were 0.6. V2 did not show a clear influence of distribution parameters from which the data was sampled on AUCs as opposed to V1. In summary, V1 outperforms V2 also in larger datasets.

### Semi-quantitative classification and two true underlying groups

**Figure 6:**
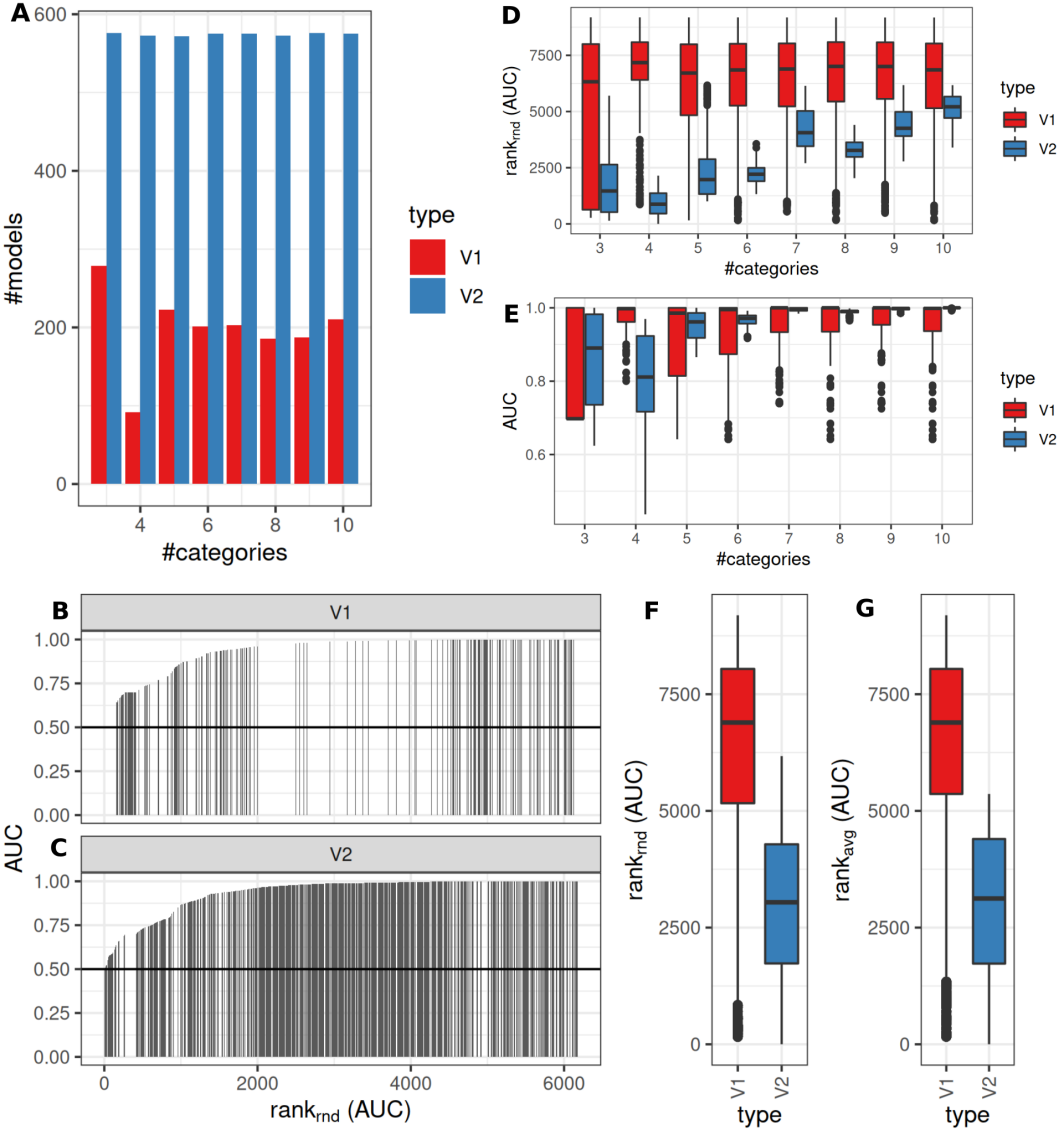
Comparison of feature preselection methods for semi-quantitative equidistant classification with true dichotomous underlying groups. (A) Number of successfully trained models (Benjamini-Hochberg multiplicity adjustment). AUCs (B–C) and AUC ranks (F–G) ranks of V1 and V2, rank-ties methods: avg, average, rnd, random. (D) AUCs and ranks (E) split by numbers of semi quantitative categories.

An intermediate between the two previously analyzed conditions was evaluated next. Classification was allowed to be graduated (semi-quantitative), but the underlying grouping was assumed to be dichotomous. V1 lead to model fits of median 35% (range: 16–48%, Benjamini–Hochberg *p*-value adjustment) of successful model fits using V2 (Fig. 6A). Processing time was lower for V1 (Fig. S1). V1 yielded higher AUC ranks as compared to V2 (Fig. 6B). Minimum observed AUCs were 0.64 for V1 and 0.44 for V2. V2 yielded 5 models with AUCs < = 0.5. Stratification by numbers of categories showed higher median AUC ranks for V1 and increasing median ranks for V2 (Fig. 6C), as well as increases in AUCs for > = 4 categories for V2 (Fig. 6C). Stratification of AUCs by error probability (prob1) and number of categories showed decreases of AUCs for increasing error probabilities especially for V2 (Fig. S6). Dependency of AUCs on numbers of categories, mean difference and standard deviations between the two underlying groups showed higher AUCs for lower standard deviations especially for 4 groups for V1 (Fig. S6).

### Use case: methylation based identification of prognostically different CIMP-glioblastomas

**Figure 7:**
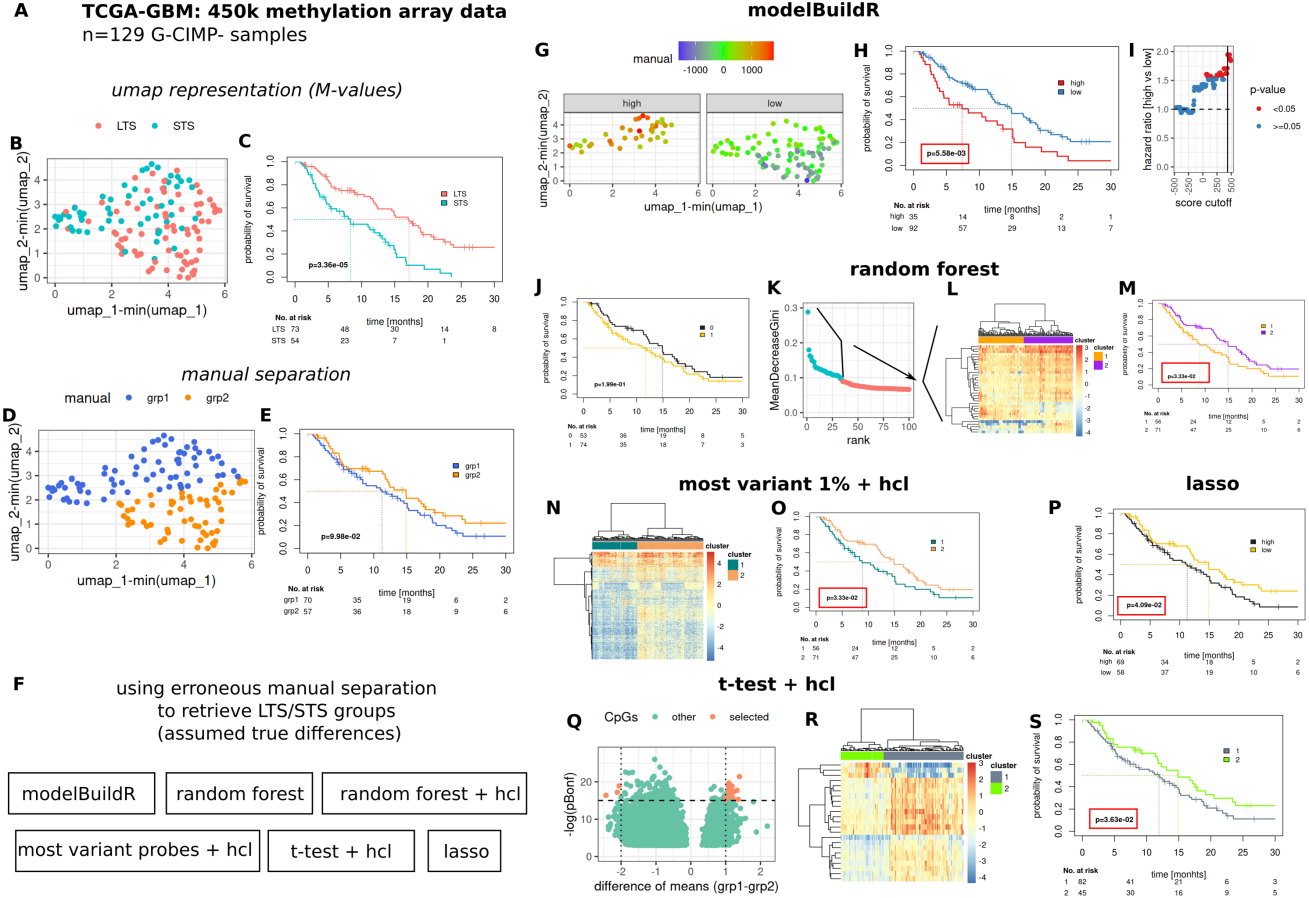
Comparative evaluation of the novel proposed heuristic to identify prognostically different G-CIMP-tumors from methylation array data. (A) Evaluated data. (B) Umap representation of M-values with LTS/STS classification (see Fig. S7 and Suppl.-Methods) and corresponding survival curves (C). Manual separation of prognostically different tumors, umap (D) and survival curves (E). (F) Evaluated approaches to detect groups of prognostically different tumors. hcl: hierarchical cluster analysis. (G–I) modelBuildR heuristic, (G) color-coded model scores in umap representation of methylation data and separated by prognostic group (high/low, see H). (H) Survival curves correspond to best achievable separation (minimal *p*-value, vertical line, I). Random forest predictions (J), random forest derived ranking of CpG probes (K, importance, mean decrease Gini), hierarchical cluster analysis of selected probes (L, blue, ward.D2, Euclidean distance), survival curves of two main clusters (M). (F) Hierarchical cluster analysis (ward.D2, Euclidean distance) of 1% of most variant probes (median absolute deviation) and corresponding survival curves of two main clusters (O). (P) Survival curves for lasso regression model predictions, analogously to I. (Q) Volcano plot of differentially regulated probes (*t*-test, Bonferroni adjustment), selected probes were used for consensus clustering (R, hcl, ward.D2, Euclidean), prognostic separation of two main clusters (S). Kaplan-Meier survival curves, likelihood ratio test p-values (Cox-PH models).

To comparatively evaluate the proposed heuristic, we assessed a number of methods for their ability to identify/retrieve two assumedly true groups of prognostically different G-CIMP- GBM tumors present in the TCGA-GBM 450k methylation array data cohort (Fig. 7). Two prognostically different groups (long-term survivors, LTS and short-term survivors, STS) were defined as outlined in Fig. S7 and Suppl. Methods. An umap representation was calculated from methylation array data, distribution of LTS/STS samples is shown in Fig. 7A. LTS tumors are rather located in the lower right part, STS tumor in the upper part of the graph. Data-driven separation of samples, based on the umap representation, was performed manually with a straight line (Fig. 7B). The resulting grouping of samples (above, below the line, grp1 and grp2) was used to train a random forest classifier, a lasso regression and a logistic regression with the proposed heuristic. For the random forest classifier, an additional analysis was performed by selecting the highest ranked CpG probes (importance, mean decrease Gini, Fig. 7E, 2nd to 4th column) for subsequent hierarchical cluster analysis. Additional methods for feature selection are shown in Figs. 7F–7H. Predictions from the random forest classifier, two main clusters for approaches involving hierarchical [consensus] clustering and optimal prognostic separation of continuous values (predictions from lasso and the novel heuristic, minimum *p*-values) were compared w.r.t. their ability for prognostic separation (Figs. 7D–7H and Table 1). Best separation was achieved with the novel heuristic (median survival difference of 7.51 months), followed by random forest classifier with hierarchical cluster analysis of most important CpGs (6.04 months). Selection of BIC, AIC or CV approach in fitM() yielded the same model (Table S1).

## Discussion

Omics-data, e.g., expression or methylation data is often used to gain insights the underlying biology (Capper et al., 2018). In translational research, molecular data from patients is often compared against a given binary classification (e.g., tumor subtype A vs B) or a graduated semi-quantitative rating, e.g., of an immune-histochemical straining of intensity classes 1 to 5 (Balermpas et al., 2017; Knoll et al., 2016). However, prospectively measured inter-rater agreement for classification of grade and histotype of ovarian cancer by specialists (pathologists) has been reported with only 85–95% (Barnard et al., 2018). Thus, a fraction of misclassifications of 5–15% might be considered a conservative estimation even for trained raters.

**Table 1:** Comparison of different methods for prognostic separation of G-CIMP negative glioblastoma tumors based on methylation array data.

Method	Median survival difference [months]	HR, 95% CI	*p*-value
Random Forest	3.2	1.3 [0.86–2.03]	0.2
Random Forest + hcl	6.04	0.5 [0.41–0.96]	0.03
Most variant + hcl	4.5	0.8 [0.54–1.30]	0.4
*t*-test + hcl*	3.0	0.6 [0.39–0.98]	0.04
Lasso	3.7	0.6 [0.42–0.99]	0.04
modelBuildR	7.51	0.5 [0.33–0.81]	0.004

**Notes.**

Hcl hierarchical cluster analysis * consensus clustering HR hazard ratio, Cox-PH models

Model training for classification might hint towards underlying biological mechanisms as the model training step is assumed to select features which robustly allow to infer groups. Evaluation of all possible combinations of features for model training is not feasible for typical datasets and for standard modelling approaches also often not possible (numbers of features > > numbers of samples), ridge regression and lasso (Santosa & Symes, 1986; Tibshirani, 1996) allow to deal with such data. Alternatively, features can be pre filtered with a wide variety of methods (Lazar et al., 2012). Binary outcomes can be modeled with logistic regressions and graduated, equidistant classifications with linear models. For a discussion of currently applied methods for the analysis of real-world clinical data—starting from simple ROC based analyses to complex models and feature selection approaches—see Chen et al. (2019) and Deo (2015). Even though deep-learning models might show extraordinary high performance for specific tasks in biomedical research, their application if often limited by sparsity of data or low quality (Chen et al., 2019) and might be vulnerable to small adversarial perturbations (Yuan et al., 2019). Therefore, novel methods are needed with specifically enable analysis using poorer quality data. Furthermore, highly complex and powerful deep-learning methods lack transparency (Holzinger et al., 2019), but explainability and interpretability often is a crucial point needed to gain a more mechanistic understanding of underlying processes.

Methods often aim to explain the observed data (classification) as good as possible, e.g., by using goodness of fit tests or sufficient differences in information criteria, while addressing overfitting e.g., by incorporating cross-validation. However, selection of features is still based on (probable) erroneous classification.

We aimed to evaluate if a heuristic which inverts the roles of dependent (classification) and independent (features) variables in a pre-filtering step might help to retrieve features associated with the true underlying structure/grouping (testing for significant differences, cross validation). Therefore, we simulated data for two or more distinct classes, added an error on the classification and tried to retrieve the original classification as quantified by (multiclass) ROC analyses.

Evaluation of true different populations encoded semi-quantitatively showed no global preference for V1 or V2 except for lower time requirements for V1. AUC ranks were still higher for V1, thus making V1 a reasonable analysis approach. The presence of only small differences between populations, however, might impair performance in this setting.

The presence of two true groups can be encoded binary by a (human) rater or, e.g., for immune histochemical stainings, graduated even though only two groups are present. Both combinations were evaluated, showing a clear overall benefit of the proposed heuristic for binary encodings. This was not only true for systematic analyses with few numbers of features (*n* = 100) and 25 samples per group, but also for larger datasets with up to 10,000 features and 100 samples per group. For semi-quantitative encodings, a better performance was seen for lower numbers of semi-quantitative categories. Thus the heuristic can be recommended for binary classified data, and if only few categories (∼4) are used for classification if a binary ground truth might be present. Due to large time requirements, only the combination yielding a clear benefit (two groups, binary classification), was tested with larger numbers of features and samples.

The proposed heuristic for feature pre-filtering leads to a number of combinations where no model could be fit. These combinations, however, would have led to models with low AUCs (compared to the ground truth) using the cross validation only feature selection strategy (V2). More liberal *p*-value adjustment strategies were not always beneficial, thus performance of different multiplicity adjustment procedures with varying *p*-value cutoffs while considering their respective power should be evaluated in future work. Lower time-requirements of the heuristic might prove useful especially for larger datasets.

We utilized the TCGA-GBM 450k methylation array data cohort of G-CIMP negative tumors to demonstrate the ability of the proposed heuristic to retrieve features able to separate probable true different underlying groups of tumors. Direct comparison with additional methods, even for only a small number of approaches, showed a superior performance of the novel heuristic. Without interpreting too much into the potential biological meaning (no independent validation), it is worth noting that methylation array data is used to detect and classify separate subgroups of glioma and G-CIMP- glioblastoma, which also show differences in prognosis (Capper et al., 2018; Knoll et al., 2019; Hwang et al., 2019).

In summary, the proposed heuristic proved most beneficial for the identification of two groups encoded in two or few categories. Identified features were then more probable to represent true associated characteristics. However, future work is needed to validate these findings in more complex/real-world data with e.g., unbalanced groups, larger sample sizes and multiple (non-)correlated true effects in underlying data. For an easy application of such benchmarks, our modelBuildR package can be used and is made publicly available on github.

## Conclusions

In biomedical research, misclassification is not negligible with reported error rates up to 15%. Classical feature selection methods, however, assume that a provided labeling is correct and select features best explaining potentially erroneous data, even though interest lies in true underlying groups. We propose a novel feature selection heuristic which inverts roles of dependent and independent variables in an initial feature selection step and proceeds with standard methods. Its superior performance in identifying features associated with the ground truth even for wrongly labeled samples is demonstrated in synthetic data arising from two true groups and binary manual encoding. A use case with methylation array omics data shows promising results. Further work is needed to better characterize applications for which the proposed heuristic might be beneficial.

##  Supplemental Information

10.7717/peerj.10849/supp-1 Figure S1 Time requirements for different evaluated datasets

10.7717/peerj.10849/supp-2 Figure S2 AUC stratified by parameters used for simulation of data with semi-quantitative classification and graduated ground truth BH *p*-value adjustment in prefiltering step.

10.7717/peerj.10849/supp-3 Figure S3 AUC stratified by parameters used for simulation of data with semi-quantitative classification and graduated ground truth Bonferroni *p*-value adjustment in prefiltering step.

10.7717/peerj.10849/supp-4 Figure S4 AUC stratified by parameters used for simulation of data for binary classification and dichotomous ground truth BH *p*-value adjustment in prefiltering step.

10.7717/peerj.10849/supp-5 Figure S5 AUC stratified by parameters used for simulation of data for binary classification and dichotomous ground truth Bonferroni *p*-value adjustment inprefiltering step and numbers of models with AUC < = 0.5.

10.7717/peerj.10849/supp-6 Figure S6 AUC stratified by parameters used for simulation of data with semi-quantitative classification and dichotomous ground truth BH *p*-value adjustment in prefiltering step, data shown for 3,4 and 5 groups.

10.7717/peerj.10849/supp-7 Figure S7 Comparison of feature presentation methods for binary outcomes with underlying true dichotomous groups and for larger number of features and samples (A) AUCs of model predictions tested against group truth for varying classification probabilities, numbers of samples and features. (B) AUC ranks for V1 and V2, rank-ties methods: avg average, rnd: random. (C) AUCs stratified by parameters used for simulation of data. Bonferroni p-value adjustment.

10.7717/peerj.10849/supp-8 Figure S8 Identification of prognostically different tumors and associated differentially methylated probes from the TCGA-GBM 450k methylation array dataset Kaplan–Meier survival curves, Likelihood Ratio Test (Cox-PH) *p*-values. See Suppl-Methods for details.

10.7717/peerj.10849/supp-9 Table S1 modelBuildR dervied model for prognostic separation of G-CIMP- tumors from the TCGA-GBM cohort M-values were used for model training, binary outcome (grp1 vs grp2, Fig. 7B), default parameters.

10.7717/peerj.10849/supp-10 Supplemental Information 1 Supplemental Methods

## Additional Information and Declarations

### Data Availability

The following information was supplied regarding data availability: The R package is available at Github: http://github.com/mknoll/modelBuildR. Code for the simulation study are accessible at CodeOcean: Maximilian Knoll (2020) modelBuildR: An R package for model building and feature selection with erroneous classifications. [Source Code]. https://doi.org/10.24433/CO.3805663.v1.
