# Supplementary figures and images for: modelBuildR: an R package for model building and feature selection with erroneous classifications
Source: PeerJ. 2021 Feb 9;9:e10849. doi: 10.7717/peerj.10849 (PMC7879945; doi:10.7717/peerj.10849)

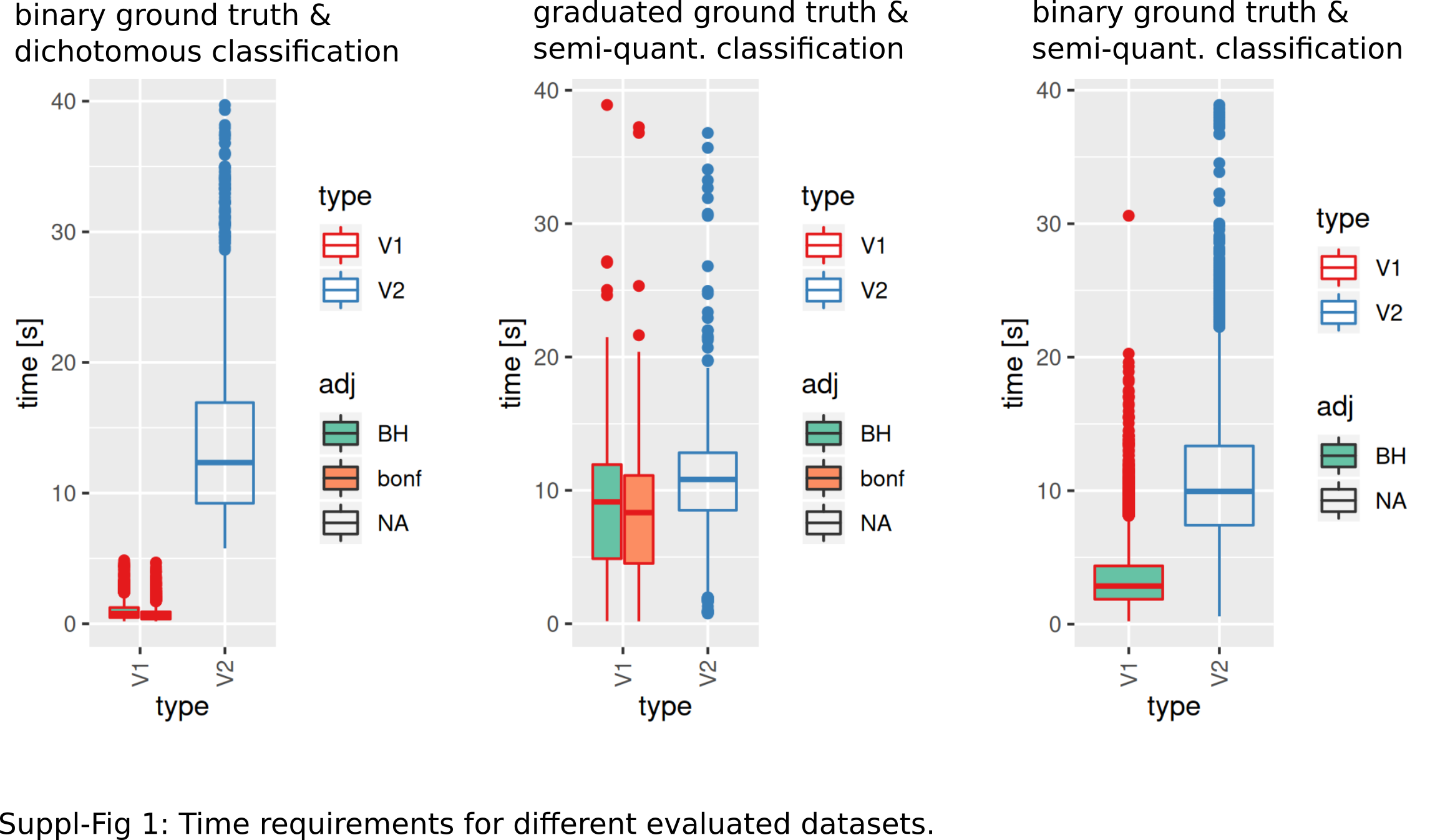

Supplement: Figure S1 [file peerj-09-10849-s001.png]

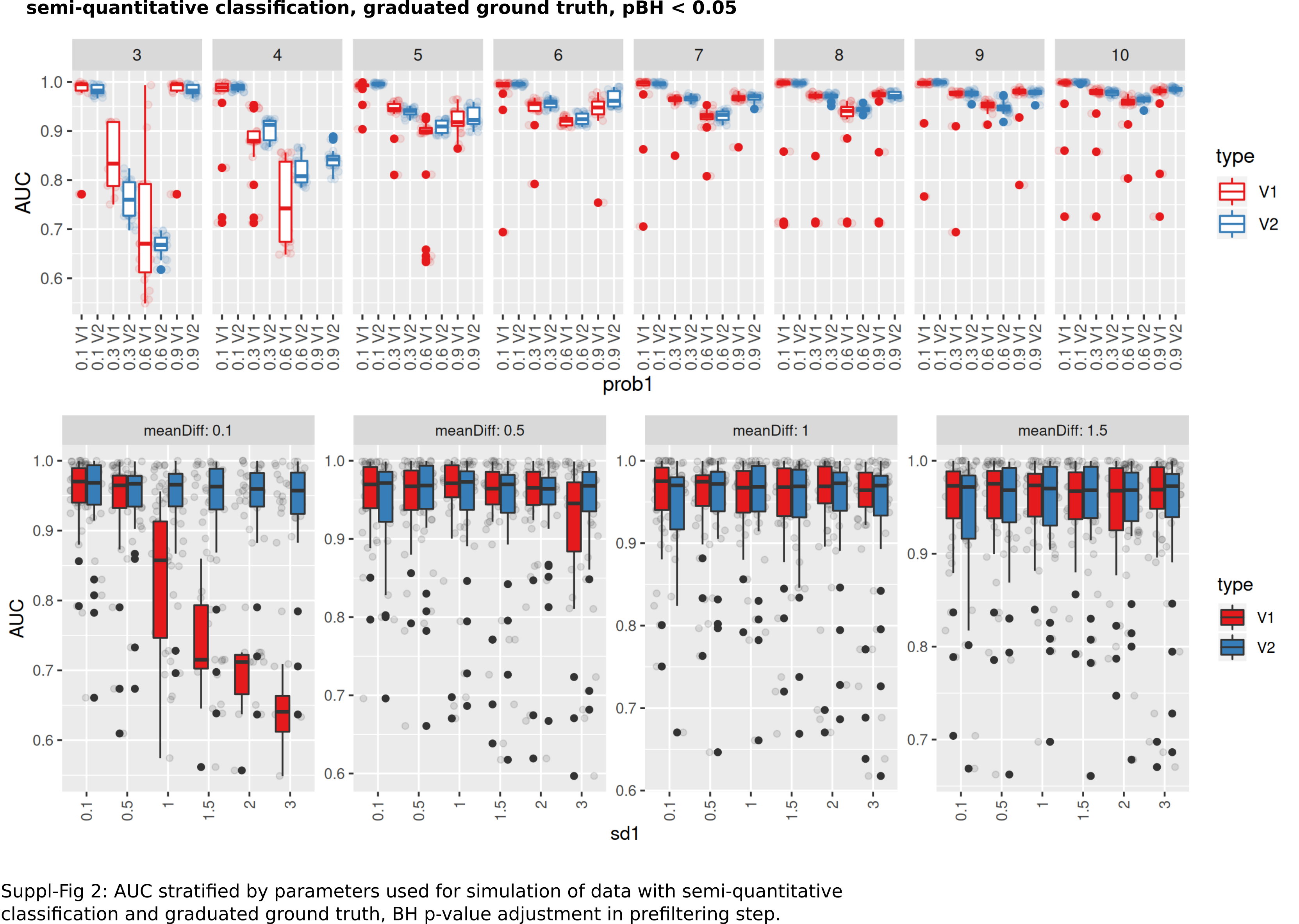

Supplement: Figure S2 — BH p-value adjustment in prefiltering step. [file peerj-09-10849-s002.png]

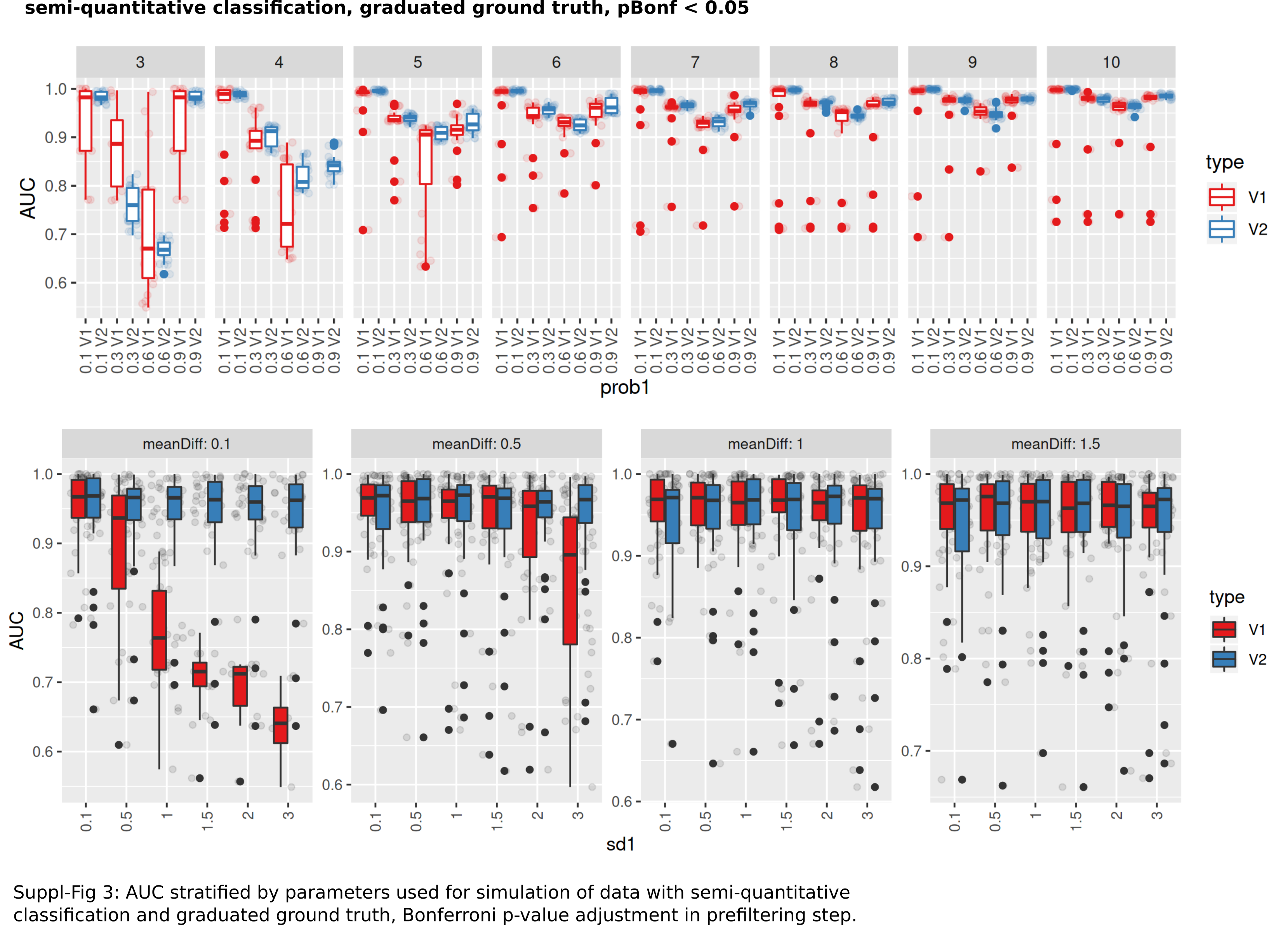

Supplement: Figure S3 — Bonferroni p-value adjustment in prefiltering step. [file peerj-09-10849-s003.png]

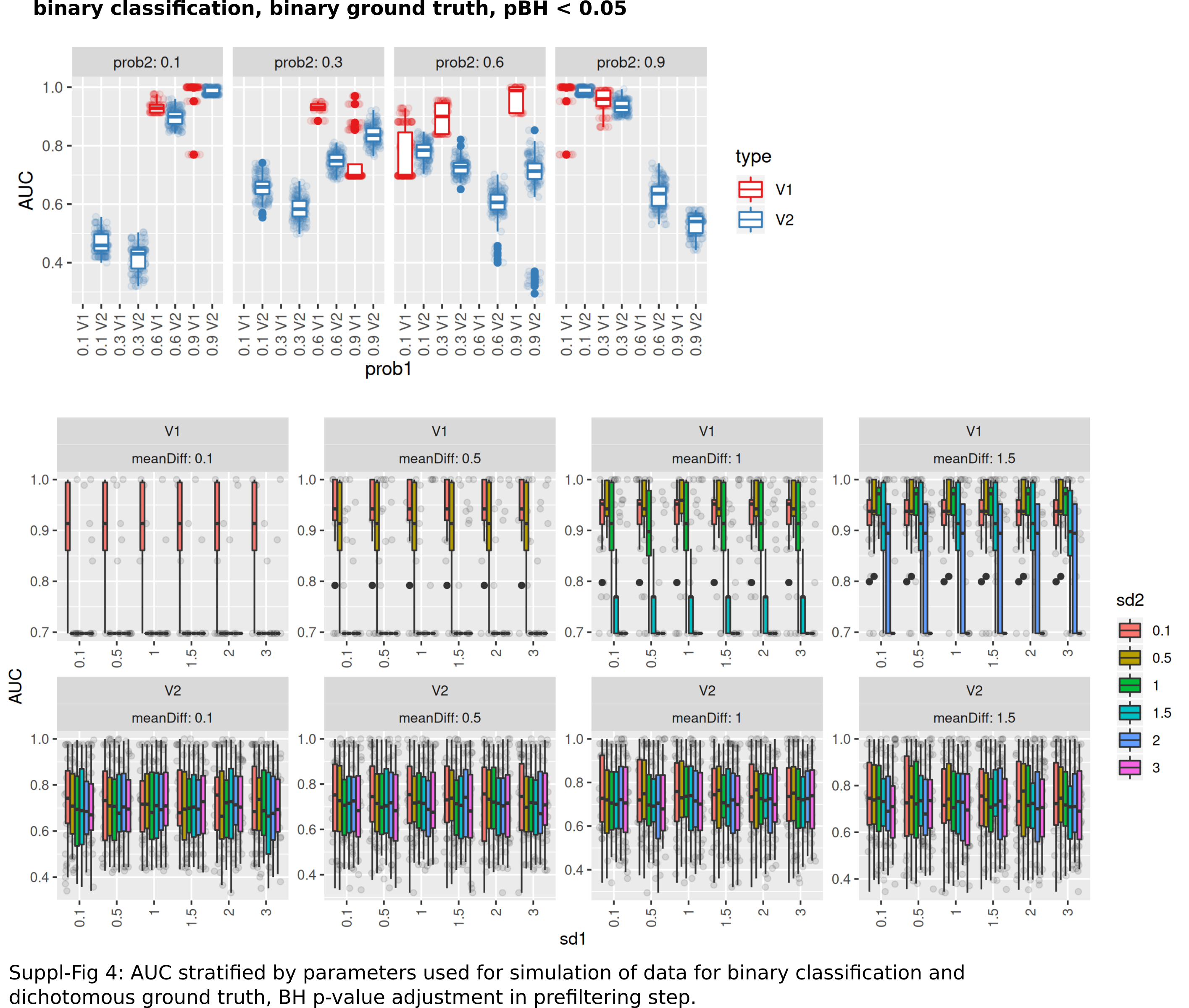

Supplement: Figure S4 — BH p-value adjustment in prefiltering step. [file peerj-09-10849-s004.png]

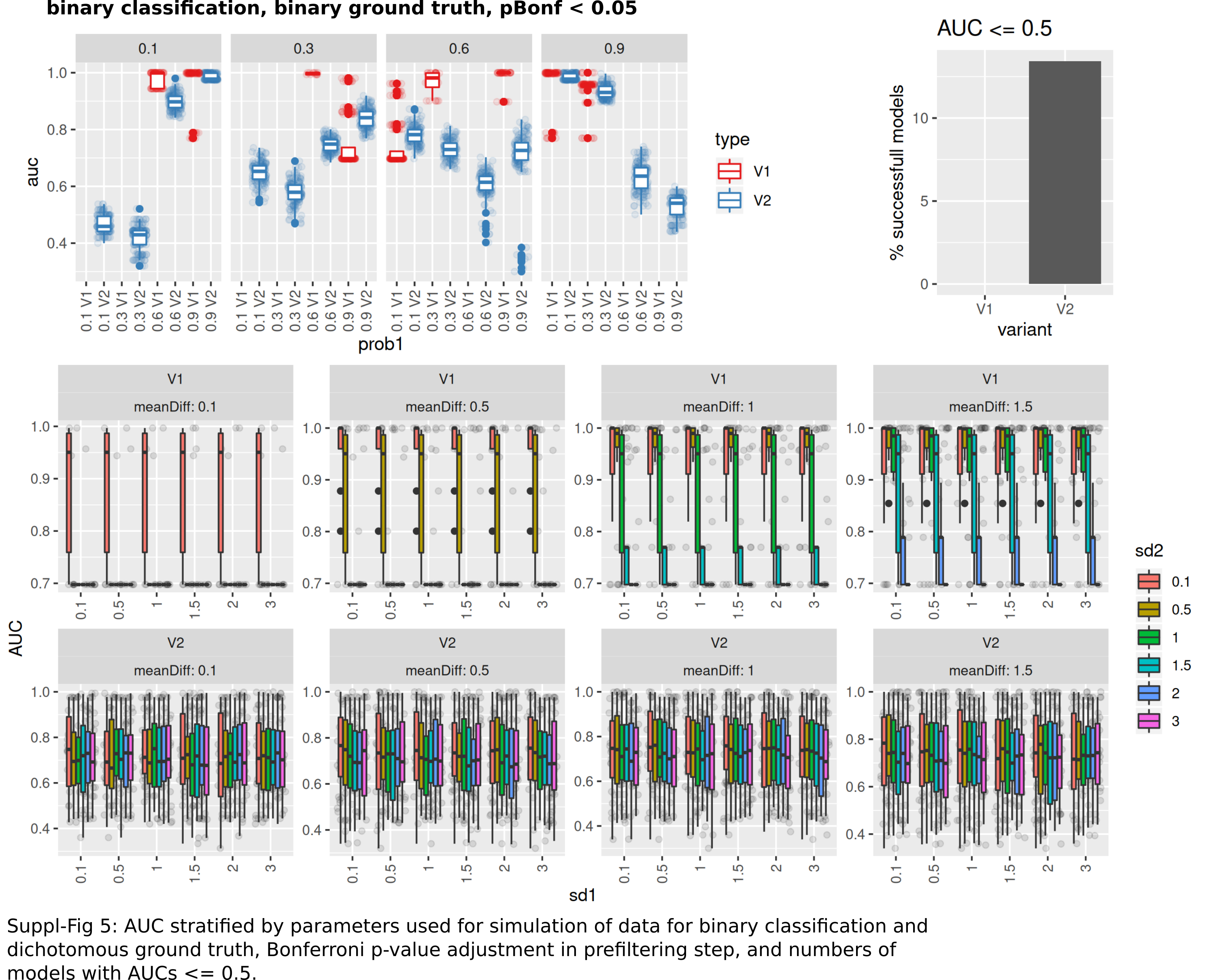

Supplement: Figure S5 — Bonferroni p-value adjustment inprefiltering step and numbers of models with AUC < = 0.5. [file peerj-09-10849-s005.png]

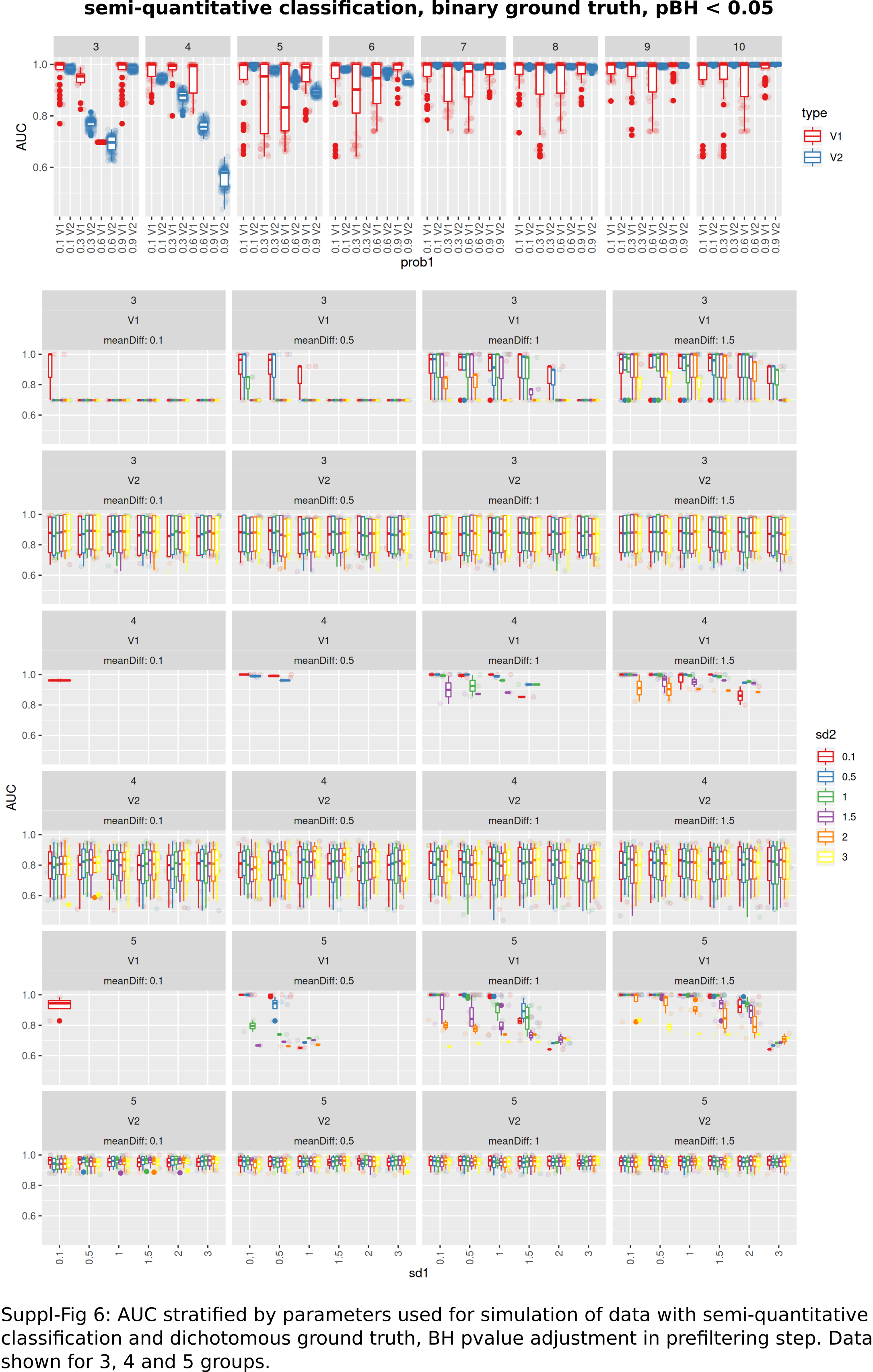

Supplement: Figure S6 — BH p-value adjustment in prefiltering step, data shown for 3,4 and 5 groups. [file peerj-09-10849-s006.png]

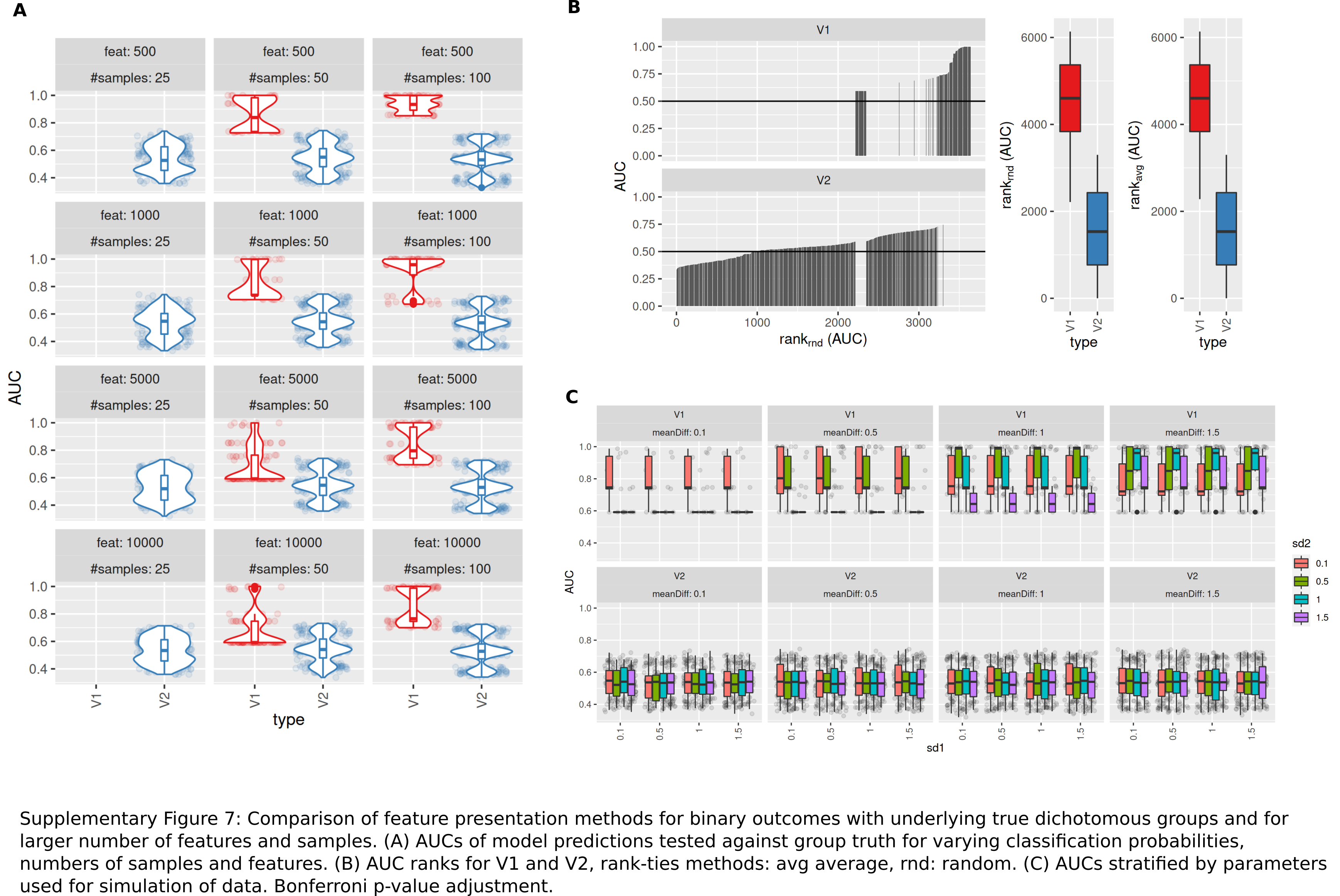

Supplement: Figure S7 — (A) AUCs of model predictions tested against group truth for varying classification probabilities, numbers of samples and features. (B) AUC ranks for V1 and V2, rank-ties methods: avg average, rnd: random. (C) AUCs stratified by parameters used for simulation of data. Bonferroni p-value adjustment. [file peerj-09-10849-s007.png]

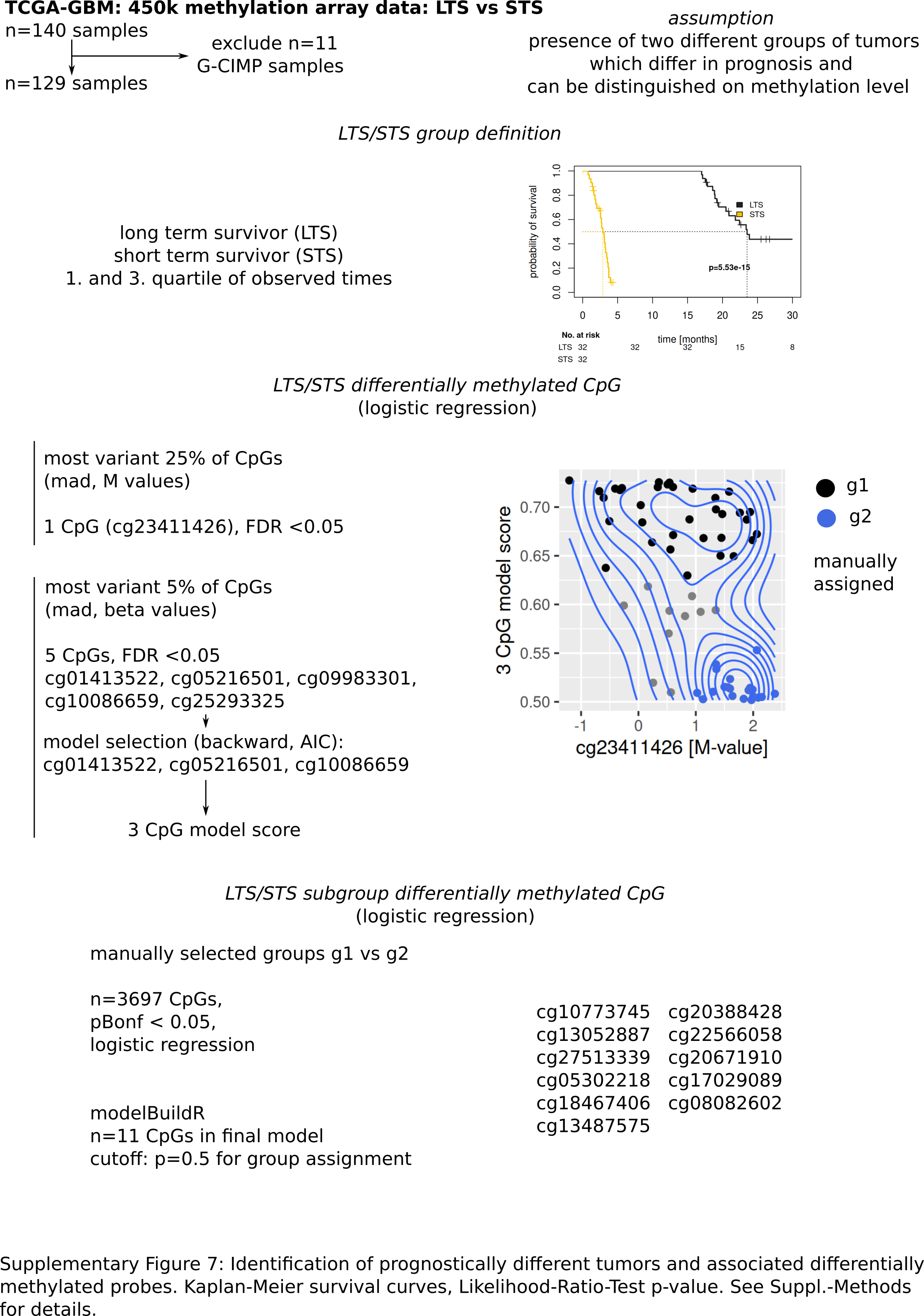

Supplement: Figure S8 — Kaplan–Meier survival curves, Likelihood Ratio Test (Cox-PH) p-values. See Suppl-Methods for details. [file peerj-09-10849-s008.png]
