# Supplementary material for: modelBuildR: an R package for model building and feature selection with erroneous classifications
Source: PeerJ. 2021 Feb 9;9:e10849. doi: 10.7717/peerj.10849 (PMC7879945; doi:10.7717/peerj.10849)
Supplement: Supplemental Information 1 [file peerj-09-10849-s010.docx]

## Identification and assignment of LTS/STS tumors

On overview of the different steps for identification of LTS/STS tumors is shown in Suppl.-Fig. 7. First, all G-CIMP+ tumors were removed from the TCGA-GBM dataset (n=11), retaining 129 samples for further analysis. It was assumed that two groups of CIMP- tumors were present in the datasets, differing in their prognosis and on epigenetic level. First, all observed survival timed (neglecting censoring status) were evaluated, samples with values below the first quartile were labeled as short-term-survivor (STS), above the third quartile as long-term survivor (LTS).

To detect differential methylation profiles, the most variant 25% of CpGs (median absolute deviation of M values) were evaluated for differentially methylated probes, identifying cg23411426 with an FDR < 0.05, logistic regression, LTS/STS as dependent variable. Next, the most variant 5% of probes (median absolute deviation, beta values) were evaluated analogously, yielding five differentially methylated CpGs. Backward, AIC based model selection retained three out of these (cg01413522, cg05216501, cg10086659). Logistic model predictions of this model was logit^-1 transformed, yielding the 3 CpG model score. Both the score value and cg23411426 M-values were plotted, areas of high density were manually defined as intermediate groups (g1, g2).

The modelBuildR heuristic was used to derive a model for classification of these groups (3697differentially methylated CpGs, pBonf < 0.05, t-test), which was then used to assign groups to all samples.

## Model selection using cross validation

Pseudocode for the two cases (binary outcome / continuous outcome) are shown below. Note that this method retains at least one feature. Threshold parameters can be adjusted and are used to detect a steep decrease in accuracy (stop criterion logistic regression) or a uniform cross validation error distribution over features (stop criterion linear model).

### logistic regression

Binary dependent variable: 0, 1

features #previously selected features

maxCVAccuracy #maximum cv accuracy for each iteration and full model

fitB #currently selected model

threshold = 0.9

fit #fit model with all features

calculate crossvalidation accuracy with DAAG::cv.binary and store in maxCVAccuracy

fitB = fit

while (true) {

for (feature in features) {

fit model with all features except feature

calculate crossvalidation accuracy with DAAG::cv.binary

}

minAccFeat = feature with minimum accuracy from previous for loop

fit = model with all but minAccFeat

curMaxAc = maximum observed accuracy from this iteration

if (curMaxAc < threshold*max(maxCVAccuracy)) {

stop, as accuracy of the new model is too low

}

fitB = fit

remove minAccFeat from features

if (number of remaining features == 1) {

stop

}

}

return fitB

### linear model

Continuous dependent variable

features #previously selected features

maxMS #maximum ms for each iteration and full model

fitB #currently selected model

threshold = 0.75

fit #fit model with all features

calculate crossvalidation error with DAAG::cv.lm and store in maxMS

fitB = fit

while (true) {

for (feature in features) {

fit model with all features except feature

calculate crossvalidation error with DAAG::cv.lm

}

maxMSFeat = feature with maximum crossvalidation error from previous for loop

fit = model with all but maxMSFeat

curMaxMS = maximum observed crossvalidation error from this iteration

if (curMaxMS < threshold*max(maxMS)) {

stop, as CV error decrease is not large enough anymore

}

fitB = fit

remove maxMSFeat from features

if (number of remaining features == 1) {

stop

}

}

return fitB
